# Plasma-Derived Exosomes from NAFLD Patients Modulate the Cannabinoid Receptors’ Expression in Cultured HepaRG Cells

**DOI:** 10.3390/ijms24021739

**Published:** 2023-01-16

**Authors:** Valentina De Nunzio, Livianna Carrieri, Maria Principia Scavo, Tamara Lippolis, Miriam Cofano, Giusy Rita Caponio, Valeria Tutino, Federica Rizzi, Nicoletta Depalo, Alberto Ruben Osella, Maria Notarnicola

**Affiliations:** 1 Laboratory of Nutritional Biochemistry, National Institute of Gastroenterology IRCCS “S. de Bellis” Research Hospital, 70013 Castellana Grotte, Italy; 2 Laboratory of Personalized Medicine, National Institute of Gastroenterology IRCCS “S. de Bellis” Research Hospital, 70013 Castellana Grotte, Italy; 3 Dipartimento di Chimica, Università degli Studi di Bari Aldo Moro, Via Orabona 4, 70125 Bari, Italy; 4 Institute for Chemical-Physical Processes (IPCF), Council National Research (CNR) Bari, Via Orabona 4, 70125 Bari, Italy; 5 Laboratory of Epidemiolgy and Biostatistics, National Institute of Gastroenterology IRCCS “S. de Bellis” Research Hospital, 70013 Castellana Grotte, Italy

**Keywords:** nonalcoholic fatty liver disease, exosome, cannabinoid receptors

## Abstract

Exosomes produced by hepatocytes upon lipotoxic insult play a relevant role in pathogenesis of nonalcoholic fatty liver disease (NAFLD), suggesting an inflammatory response by the activation of monocytes and macrophages and accelerating the disease progression. In the pathogenesis of NAFLD and liver fibrosis, the endogenous cannabinoids and their major receptors CB1 and CB2 appear to be highly involved. This study aimed at evaluating the expression of cannabinoids receptors (CB1R and CB2R) in plasma-derived exosomes extracted from patients with NAFLD, as well as investigating the in vitro effects of the circulating exosomes in cultured human HepaRG cells following their introduction into the culture medium. The results demonstrated that plasma-derived exosomes from NAFLD patients are vehicles for the transport of CB1R and are able to modulate CB receptors’ expression in HepaRG cells. In particular, circulating exosomes from NAFLD patients are inflammatory drivers for HepaRG cells, acting through CB1R activation and the downregulation of CB2R. Moreover, CB1R upregulation was associated with increased expression levels of PPAR-γ, a well-known mediator of liver tissue injury. In conclusion, this study provides evidence for CB1R transport by exosomes and suggests that the in vitro effects of circulating exosomes from NAFLD patients are mediated by the expression of cannabinoid receptors.

## 1. Introduction

Exosomes—small extracellular vesicles with a notable role in autocrine and paracrine signaling—are involved in a pivotal role in intracellular communication. Several studies implicated exosomes in various diseases and metabolic processes, including immune, inflammation, and cancer processes [1,2,3].

Exosomes are also involved in cell–cell communication both in the liver and interorgan crosstalk, promoting inflammation, immune modulation, fibrosis, and angiogenesis [3]. Moreover, it has been proven that exosomes are able to deliver their bioactive cargo (RNA, DNA, proteins, and lipids) from cell of origin to target cells and that their expression seems to be correlated with the worsening of liver damage [4,5].

Much evidence has reported that exosomes produced by hepatocytes are major contributors to triggering the inflammatory response in liver disease [6,7,8]. As extracellular nanovesicles, exosomes range in size from 30 to 150 nm and are released into the extracellular space from hepatocytes upon lipotoxic insult, carry cargoes including proteins, lipids, and microRNA, and they play a relevant role in the pathogenesis of nonalcoholic fatty liver disease (NAFLD), suggesting an inflammatory response by the activation of monocytes and macrophages and accelerating the disease progression [7,9]. Of note, NAFLD, the most common metabolism-associated fatty liver disease, is mainly involved in liver injury [9,10,11]. As previously demonstrated, the analysis of fatty acid profiles in cell membranes is important for diagnosing the disease and identifying its staging [12,13]. Recently, Stearoyl-CoA desaturase-1(SCD-1), a central lipogenic enzyme for the synthesis of monounsaturated fatty acids, known also as oleic acid/stearic acid ratio, was positively associated with the hepatocarcinoma development in mice and rats [14]. Furthermore, the understanding of NAFLD can be studied in exosomes. Scavo et al. observed the presence of the Wnt/Frizzled receptor (FZD) protein, namely, FZD 7—a G protein-coupled receptor—correlated with more severe degree of liver disease in plasma-derived exosomes of NAFLD patients [15].

A growing body of evidence highlighted that exosomes are the major contributors in initiating inflammation in the liver [16]. Experimental mice and rat models of hepatic disease have showed a significant increase in the plasma extracellular vesicles’ levels, suggesting that these microvesicles are mainly involved in accelerating the disease progression [17].

In the pathogenesis of NAFLD, an important role has been attributed to the EC system [18], which comprises endogenous cannabinoids, their receptors (CB1R and CB2R), and the enzymes responsible for their synthesis and degradation. This system affects the molecular mechanisms sustaining hepatocytes injury and the inflammatory response into liver tissue. During acute and chronic liver damage, the expression of CB1R has demonstrated to be significantly increased, with an increase in fibrinogenesis, whereas CB2R is downregulated, with an improved antifibrinogenic response [19]. In this context, CB2R knock-out mice seem to be more susceptible to hepatic insult, showing enhanced liver inflammation and steatosis compared to wild-type mice [19].

Furthermore, a recent study showed a functional and structural relation between the endocannabinoid (EC) system activity and G protein-coupled receptors, including FZD 7 [20].

Active endocannabinoids have been observed on extracellular membranes vesicles, demonstrating a functional role for these vesicles in the nervous system [21]. The endocannabinoids produced by microglia and carried by vesicles are able to stimulate CB1R. This evidence identifies the extracellular vesicles as biologically active molecules to modulate the adult nervous system functions.

In the liver, the molecular mechanisms by which the EC system participates in NAFLD pathophysiology are still unclear. This study aimed to evaluate the expression of CB1R and CB2R in plasma-derived exosomes of patients with NAFLD and to investigate the in vitro effects of circulating exosomes in cultured human HepaRG cells.

## 2. Results

### 2.1. Exosomes’ Isolation and Characterization

DLS, TEM analysis, and ζ-potential measurements were performed to characterize the plasma-extracted exosomes in terms of size, size distribution, morphology, and surface charge.

**Figure 1:**
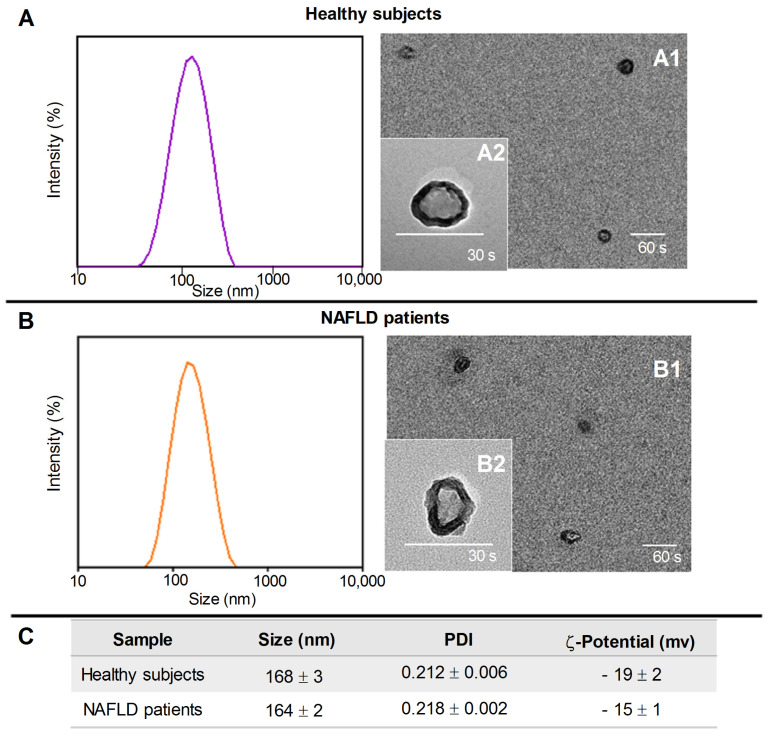
Representative DLS-intensity distribution and TEM micrographs (scale bar 200 nm) of exosomes isolated from healthy subjects (**Panel A**) and NAFLD patients (**Panel B**) at two different staining times, 30 s (A2 and B2) and 60 s (A1 and B1). (**Panel C**), the average hydrodynamic diameter and polydispersity index (PDI) obtained by DLS analysis and ζ-potential value of the exosomes (mean ± SD).

Figure 1, Panel A and B provide a monomodal size distribution of exosomes isolated from healthy subjects and NAFLD patients, respectively.

TEM micrographs indicated the isolation of bilayer-delimited nano-objects with round shape, typically ascribed to exosomes, and size in good accordance with DLS data (Figure 1, Panel A1,A2,B1,B2). Finally, the presence of a negative charge, due to the phospholipid-based membrane structure of the exosomes’ surface, was highlighted by ζ-potential measurements (Figure 1 Panel C).

### 2.2. Protein Expression of CB1, CB2 Receptors, FZD 7, and IL-1β

The analysis of CB1R and CB2R protein expression in the isolated exosomes suggested a fundamental role of exosomes in the vehiculation of these receptors.

**Figure 2:**
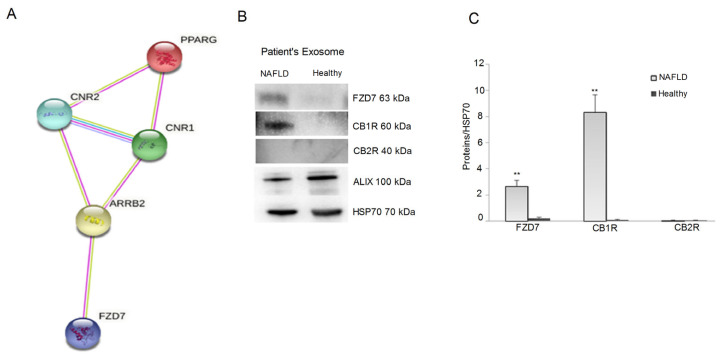
(**Panel A**), predictive analysis of the interactions between *FZD7*, *CB1R*, *CB2R*, and *PPAR-γ* genes performed by STRING database. (**Panel B**), representative blots of FZD7, CB1R, and CB2R proteins evaluated in the exosomes derived by NAFLD patients and healthy subjects. (**Panel C**), protein levels of FZD7, CB1R, and CB2R in the exosomes derived by NAFLD patients and healthy subjects. All data represent the results of three different experiments (mean ± SD). *p*-value was determined by one-way ANOVA. ** *p* < 0.001.

Moreover, in order to investigate the molecules involved in the modulation of CB receptors’ expression in the exosomes, we also considered the protein levels of FZD 7, well-known to be an upstream factor of the cannabinoid receptors. In this regard, a prediction of the interacting proteins involved in the relationship between FZD 7, CB1R, CB2R, and PPAR-γ was performed by using the STRING database. This analysis indicated that FZD 7 interacts with both CB receptor genes and, consequently, also with PPAR-γ, through the mediation of β-arrestin, promoting cell migration and proliferation [22] (Figure 2 Panel A). Based on the prediction performed using STRING, our results demonstrated the presence of CB1R and FZD7 in the exosomes derived from NAFLD patients, whereas no expression of CB2R was observed (Figure 2 Panel B,C).

**Figure 3:**
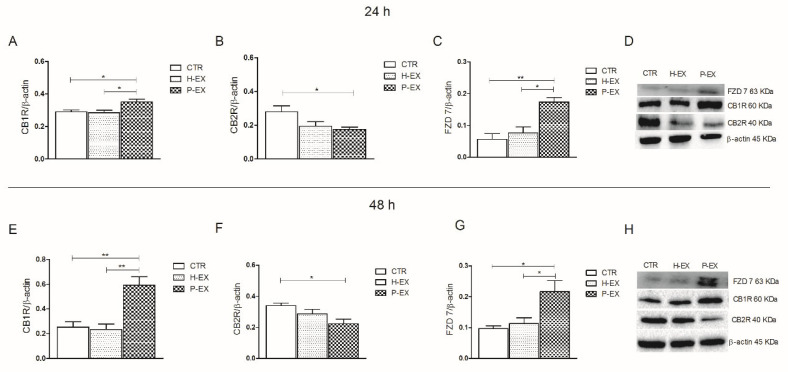
Western blotting analysis of CB1R, CB2R, and FZD7 proteins in control HepaRG cells (CTR), HepaRG cells treated with plasma-derived exosomes from healthy subjects (H-EX), and HepaRG cells treated with plasma-derived exosomes from NAFLD patients (P-EX) after 24 (**Panel A**–**C**) and 48 h (**Panel E**–**G**). (**Panel D**,**H**) show representative blots evaluated in control HepaRG cells (CTR), H-EX, and P-EX groups, after 24 and 48 h, respectively. All data represent the results of three different experiments (mean ± SD). *p*-value was determined by ANOVA and Tukey’s Multiple Comparison Test. * *p* < 0.05, and ** *p* < 0.001.

The isolated exosomes were able to affect the cellular expression of CB1R and CB2R in HepaRG cells. The cultured cells treated with exosomes of patients with NAFLD showed a significant increase in CB1R expression compared to the control and to the cells treated with exosomes derived from healthy subjects, both at 24 h and 48 h (Figure 3 Panel A,E, respectively, ANOVA and Tukey post-test, * *p* < 0.05, ** *p* < 0.001). In the same cells, CB2R expression was significantly reduced when compared to control cells and the cells treated with exosomes derived from healthy subjects, both after 24 and 48 h of treatment (Figure 3 Panel B,F, respectively, ANOVA and Tukey post-test, * *p* < 0.05). The effect of the treatment with exosomes on the expression of two CB receptors was not time-dependent. Panel C and G show the levels of protein expression of FZD 7 at 24 h and 48 h, respectively. A significant increase in FZD7 expression was observed when the cells were treated with exosomes derived from patients with NAFLD compared to the cells treated with exosomes derived from healthy subjects and to control cells (ANOVA and Tukey’s post-test, * *p* < 0.05, ** *p* < 0.001).

**Figure 4:**
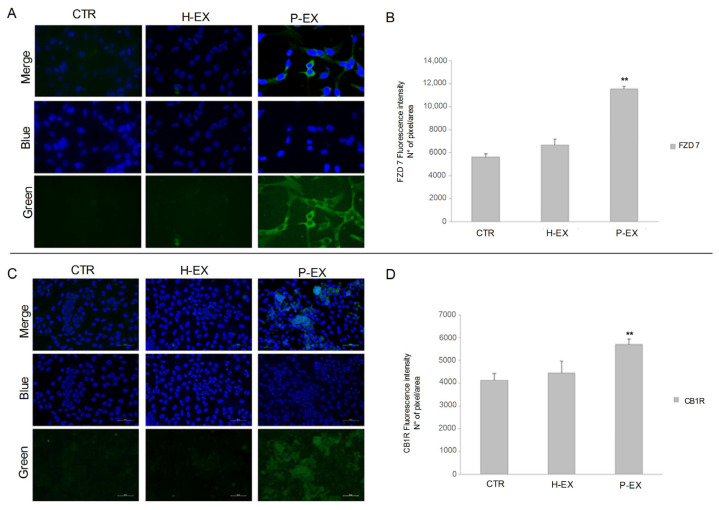
Detection of FZD 7 protein (**Panel A**) and CB1R protein (**Panel C**) by immunofluorescence confocal microscopy (20X) in control HepaRG cells (CTR), HepaRG cells treated with plasma-derived exosomes from healthy subjects (H-EX), and HepaRG cells treated with plasma-derived exosomes from NAFLD patients (P-EX) after 24 h. (**Panel B**,**D**) show a semiquantitative evaluation of FZD 7 and CB1R protein levels, respectively, in the three experimental groups. *p*-value was determined by one-way ANOVA, ** *p* < 0.001.

Immunofluorescence imaging was also performed to monitor changes in the FZD 7 and CB1R proteins’ expression on treated and untreated cells. The cellular immunofluorescence images demonstrate, for each condition, a prominent increase in green fluorescence intensity for both receptors in the cells treated with exosomes derived from NAFLD patients compared to the corresponding negative controls and cells treated with exosomes derived from healthy subjects. The confocal images of HepaRG cells after treatment with exosomes derived from NAFLD patients show a significant increase (** *p* < 0.001) in the expression in terms of fluorescence level of FZD 7 and CB1R when the cells were treated with exosomes derived from NAFLD patients compared to the control cells (Figure 4 Panel A,C), reaching a double value compared to the control for FZD 7 expression and 20% more for CB1R (Figure 4 Panel B,D). On the contrary, when the cells were treated with exosomes derived from healthy subjects, the fluorescence index did not change significantly compared to the control (Figure 4 Panel A–D).

The secreted proinflammatory cytokine named IL-1β was investigated by ELISA test in the medium of cultured HepaRG cells (Table 1). In the experiments, conducted for 72 h, the control was represented by the medium derived from the untreated cells. As reported in Table 1, in the medium derived from the cells treated with exosomes extracted from the plasma of NAFLD patients, a significant increase in the IL-1β levels (** *p* < 0.001) was observed with respect to the control cells and also with respect to cells treated with the exosomes derived from healthy subjects.

### 2.3. Gene Expression, CB1, CB2 Receptor, and FZD 7

**Figure 5:**
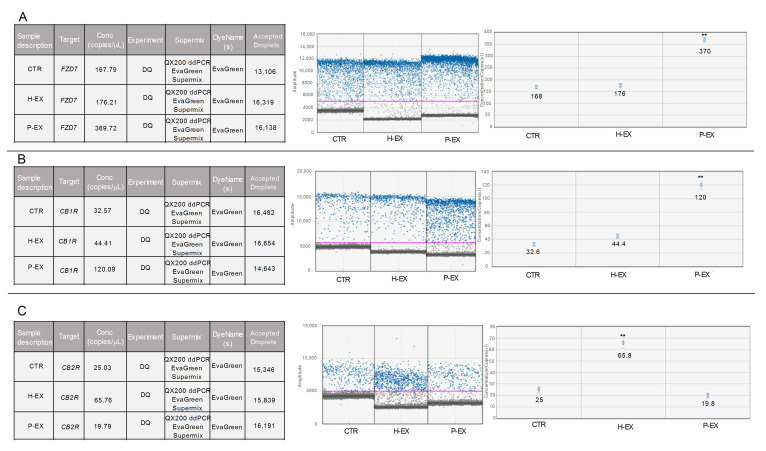
Droplet digital PCR analysis of *FZD 7* (**Panel A**), *CB1R* (**Panel B**), and *CB2R* genes (**Panel C**) in control HepaRG cells (CTR), HepaRG cells treated with plasma-derived exosomes from healthy subjects (H-EX), and HepaRG cells treated with plasma-derived exosomes from NAFLD patients (P-EX) after 6 h of treatment. The colorful lines represent the threshold value; the blue dot represents the positive samples. *p*-value was determined by one-way ANOVA, ** *p* < 0.001.

The gene expression levels of *CB1R*, *CB2R*, and *FZD 7* were evaluated by droplet digital PCR (ddPCR) in the untreated cells (CTR), in the HepaRG cells treated with exosomes derived from healthy subjects (H-EX), and HepaRG cells treated with exosomes derived from NAFLD patients (P-EX). After 6 h of the treatment, a significant increase (** *p* < 0.001) in *FZD 7* gene expression was observed (Figure 5 Panel A). Of note, a three-fold increase in gene expression was observed in exosome-treated cells from NAFLD patients compared to both control and exosome-treated cells from healthy subjects. Similarly, the *CB1R* mRNA expression levels, evaluated in the cells treated with exosomes derived from NAFLD patients, were found increased (Figure 5 Panel B). Herein, the increase was four-times higher than the untreated control (** *p* < 0.001). The investigation of *CB2R* mRNA revealed a significant increase (** *p* < 0.001) in the number of copies of mRNA in the cells treated with exosomes derived from healthy subjects with respect to the controls and, interestingly, a significant reduction of *CB2R* expression levels in the cells treated with exosomes from NAFLD patients (Figure 5 Panel C). For all genes studied, the amplitude of the actual read events was also reported in each section of Figure 5, reaching a value of above 10,000, recognized as an index of reliability. In particular, the purple line represents the threshold line between grey dots (the negative droplets) and the blue dots, which represent the read positive droplets.

### 2.4. PPAR-γ Expression

Peroxisome proliferator-activated receptors (PPARs), mainly the isoforms α and γ, are linked with the EC system, and the importance of their reciprocal regulation and modulation is well-known [23,24]. In this regard, it has been demonstrated that the pharmacological blockade or genetic ablation of CB1R affects PPAR-γ expression in the tissues [25,26]. In fact, PPAR-γ is strongly expressed in adipose tissue, but in normal conditions, the human and murine liver contain lower levels of this receptor [27]. A recent study has found a liver overexpression of PPAR-γ proteins in several murine models of obesity and type 2 diabetes mellitus [28], and this is supported by upregulation of PPAR-γ protein in a diet-induced NAFLD murine model [29].

**Figure 6:**
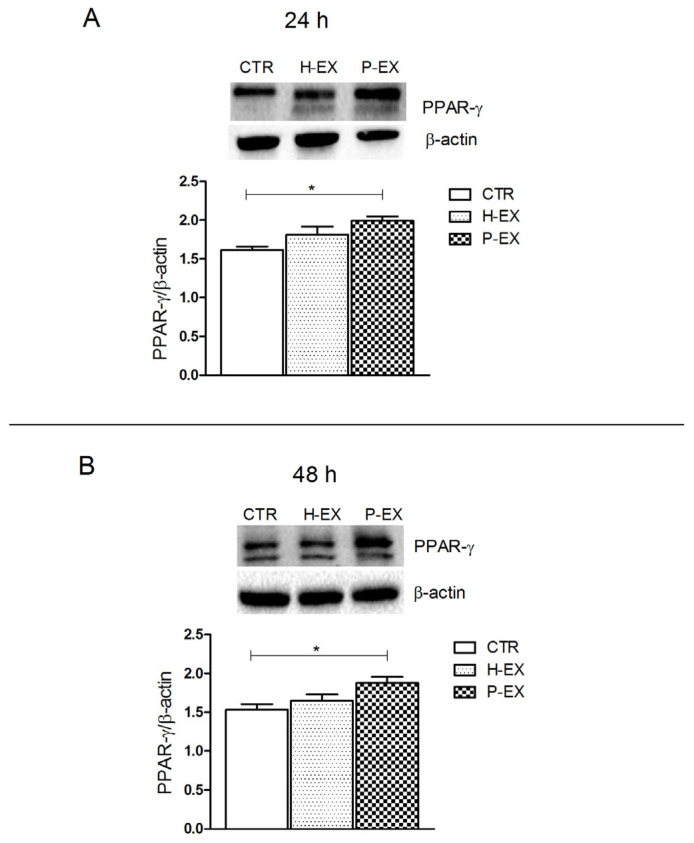
Protein levels of peroxisome proliferator-activated receptor-γ (PPAR-γ) and representative blots from control HepaRG cells (CTR), HepaRG cells treated with plasma-derived exosomes from healthy patients (H-EX), and HepaRG cells treated with plasma-derived exosomes from NAFLD patients (P-EX) after 24 h (**Panel A**) and 48 h (**Panel B**). All data represent the results of three different experiments (mean ± SD). *p*-value was determined by one-way ANOVA and Tukey’s Multiple Comparison Test. * *p* < 0.05.

In this context, here, we detected a significant overexpression (* *p* < 0.05 vs. CTR) of PPAR-γ protein after 24 and 48 h of treatment with exosomes derived from NAFLD patients compared with the control cells (Figure 6 Panel A,B, respectively).

### 2.5. Fatty Acid Analysis

The study of fatty acid profile of cell membranes is considered a valid approach to evaluate different functional aspects of a single cell. In this regard, the fatty acids’ analysis was performed both in the isolated exosomes and in cell membranes after exosome treatment. Higher levels of monounsaturated fatty acids (MUFAs) and the oleic acid/stearic acid ratio were detected both in the plasma-derived exosomes from NAFLD patients and in cell membranes treated with these plasma-exosomes (Table 2, *p* < 0.001 and *p* < 0.05). The oleic acid/stearic acid ratio is known as cellular inflammatory index and is positively associated with liver damage [30].

## 3. Discussion

Exosomes are nanovesicles released from the cells to the extracellular milieu, carrying biomolecules capable of affecting cell growth, immune responses, and cellular homeostasis [31,32]. Recently, experimental evidence has shown that exosomes and their content are actively involved in the progression of NAFLD [5]. Herein, for the first time, is reported the presence of CB1R, but not CB2R, in the plasma-derived exosomes from NAFLD patients and their ability to modulate gene and protein expression of both CB receptors and the pathway-related proteins in cultured HepaRG cells. Moreover, through a predictive biostatistics analysis, we observed an interaction between FZD 7, CB1R, CB2R, and PPAR-γ.

Several studies have demonstrated the involvement of CB receptors in acute and chronic liver injury [33,34,35], elucidating that CB1R and CB2R exert opposite effects in liver disease. CB1R is strongly upregulated in hepatocytes and during hepatic steatosis, inflammation, and liver fibrosis, whereas CB2R expression protects against liver injury because of its antifibrogenic and anti-inflammatory properties [18]. Confirming the literature data, a strong overexpression of CB1R and FZD 7 was detected in the exosomes derived from the NAFLD patients, whereas no expression of these proteins was present in the plasma-derived exosome from healthy subjects. This study also demonstrates that the regulation of gene expression of CB1R and FZD 7 begins already after 6 h from the administration of the exosomes in HepaRG cells. This result agrees with what has recently been demonstrated, showing complete absorption of exosomes and their contents within the first 4 h after administration [36]. The early cytotoxic effect of the exosomes isolated from NAFLD patients and the consequent expression of CB1R and FZD 7 in the cells determine the cellular suffering, consequently also activating PPAR-γ, a well-known mediator of liver tissue injury [37].

On the contrary, the gene and protein expression levels of CB2R in HepaRG cells treated with exosomes derived from healthy subjects were similar to the control untreated cells, confirming the protective action of this receptor in the cell. In this context, in experimental animal models, the activation of CB2R by selective agonists was demonstrated to be able to reduce inflammation and liver fibrosis [19,38].

Elevated expression of PPAR-γ has been detected in human obesity, as well as in animal models of genetic and diet-induced obesity [23] and in all clinical conditions where the EC system is overactive. PPAR-γ expression is suggestive of a tissue-specific cross-regulation, so that the ability of PPAR-γ agonists to downregulate cyclooxygenase-2 (COX-2) expression levels has been observed in breast cancer cells, macrophages, and colorectal cancer cells [26,39]. On the contrary, in liver disease, the increased levels of PPAR-γ are supportive for an inflammatory status and indicators of liver damage [23]. Furthermore, being directly activated by the CB1 receptor, PPAR-γ is able to affect metabolic homeostasis in the liver, influencing lipid metabolism. Therefore, the expression of the CB1 receptor and its downstream factor PPAR-γ influence the mechanisms responsible for cell damage and the inflammatory response differently depending on the tissue in which they act.

Recently, a dysregulation of the CB1R and PPAR-γ protein expression was observed in an animal model of intestinal bowel syndrome (IBS), in which the activation of the CB1R was associated with an improvement of mitochondrial function and gastrointestinal inflammation [40,41].

The proinflammatory effects of exosomes isolated from NAFLD patients on the cells are also due to the accumulation of specific toxic lipids inside their membranes. Recent studies have reported that high levels of palmitate in exosome membranes are responsible for modulating cellular functions that promote inflammation [42,43,44]. In the present study, the increased levels of the oleic acid/stearic acid ratio detected both in the plasma-derived exosomes from NAFLD patients and in cell membranes treated with these exosomes confirm that the oleic acid content is positively correlated with the inflammation [14,45,46].

## 4. Materials and Methods

### 4.1. Exosome Isolation

A pool of 10 plasma specimens from healthy subjects (5 males and 5 females) and a pool of 10 plasma samples from patients with severe NAFLD (5 males and 5 females) with a FibroScan^®^-diagnosed Controlled Attenuation Parameter ((CAP) ≥ 280 dB) were processed for the exosome extraction. Plasma samples were collected as part of a clinical study (N° CT02347696) and stored in the biobank of our Institute. The clinical features of the NAFLD patients were: age > 30 years and <60 years; body mass index (BMI) ≥ 25, expressed as weight in kilograms divided by the square of height in meters (kg/m2); CAP ≥ 280 dB; HOMA-IR (Homeostatic Model Assessment for Insulin Resistance) ≥ 2.5 mg/dL.

The exosomes’ isolation was performed by following the protocols previously described [47]. Briefly, venous-sampled blood specimens were kept at room temperature for 30 min and then centrifuged at 4 °C for 10 min at 1500× *g* in ethylenediaminetetraacetic acid (EDTA). The plasma was transferred to a clean tube and centrifuged again at 1800× *g* for 10 min at 4 °C; then, the supernatants were transferred into screwcap cryovial. Plasma obtained from each subject was divided into aliquots of 500 μL and frozen at −80 °C when not immediately processed. The plasma sample was centrifuged at 3000× *g* for 15 min at 4 °C, and after transferring the supernatant into a clean tube, it was centrifuged at 3800× *g* for 15 min at 4 °C. Afterward, the plasma was ultracentrifuged (BECKMAN, L-60 Ultracentrifuge) at 75,000× *g* for 1 h at 4 °C, and the resulting supernatant was transferred into another clean ultracentrifuge tube. Exosomes, obtained after a second ultracentrifugation cycle, were collected as pellet and diluted in 200 μL of ultrapure water. For each sample, 50 μL of exosome suspension was immediately processed for their characterization or cell treatment.

### 4.2. Dynamic Light Scattering (DLS) and ζ-Potential Analysis

The exosome characterization in terms of size distribution, average hydrodynamic diameter, and colloidal stability was performed by using a Zetasizer Nano ZS, Malvern Instruments Ltd., Worcestershire, UK (DTS 5.00), as previously reported [48].

### 4.3. Transmission Electronic Microscopy (TEM) Analysis

Exosomes were characterized by TEM using a Jeol JEM-1011 microscope at an accelerating voltage of 100 kV. TEM images were acquired using an Olympus Quemesa camera (11 Mpx) [47]. 

### 4.4. Cell cultures and Exosomes Administration

Human HepaRG hepatocyte cell line was purchased from Thermo Fisher Scientific, Waltham, Massachusetts, Stati Uniti and cultivated according to retailer protocols, using a hepatocyte bullet kit medium added with 10% FBS depleted of exosomes and 1% of Pen-Strep (penicillin 10,000 91 u/mL, streptomycin 10,000 u/mL, Lonza Biowhittaker, Thermo Fisher Scientific, Oslo, Norway). Cells were seeded into sterile 6-well culture plates, and when the HepaRG cells reached the confluence, they were further incubated for 24 and 48 h with a pool of exosomes derived from healthy subjects or NAFLD patients, using a total protein concentration of 20 μg/μL.

### 4.5. Western Blotting Analysis

Western blotting analysis was performed to evaluate the protein expression of FZD 7, CB1R, and CB2R in exosomes extracted from plasma from healthy subjects and from patients with NAFLD for their characterization as well as to investigate the expression of these proteins in the treated HepaRG human hepatocyte cells. Furthermore, the level of peroxisome proliferator-activated receptor-gamma (PPAR-γ), a downstream factor of the CB1 receptor pathway, was evaluated. After 24 and 48 h of cell treatment, the cells were lysed with Ripa buffer supplemented with protease and phosphatase inhibitors (Thermo Scientific, Rockford, IL, USA). Protein concentrations were determined by a standard Bradford assay (Bio-Rad, Milan, Italy), and aliquots of 50 μg of total protein extracts were loaded onto 12% precast polyacrylamide gels (Bio-Rad Laboratories, Milan, Italy). The gel was blotted onto a PVDF membrane (Bio-Rad Laboratories, Milan, Italy), and the proteins were tested with the following primary antibodies: FZD 7 (Abcam, Cambridge, UK), CB1R (Abcam, Cambridge, UK), CB2R (Abcam, Cambridge, UK), Alix (Cell Signaling Technology, Beverly, MA, USA), HSP70 (Cell Signaling Technology, Beverly, MA, USA), PPAR-γ (Santa Cruz Biotechnology, Dallas, TX, USA), and β-actin (Cell Signaling Technology, Beverly, MA, USA). After overnight incubation, the membranes were incubated with a secondary antibody, and subsequently, proteins were detected by chemiluminescence (ECL, Thermo Scientific, Rockford, IL, USA). The protein-related signal was obtained using the ChemidocTM Molecular Imager (Bio-Rad, Milan, Italy) and normalized against β-actin expression. The images were analyzed by using Image Lab 5.2.1 software.

### 4.6. Immunofluorescence Assay

HepaRG cells were seeded into eight-well slides chambers at a density of 8 × 10^3^ cells per well at 37 °C and treated, respectively, with exosomes derived from healthy subjects and NAFLD patients for 24 h. Untreated cells were used as control. The treated and untreated cells were washed with PBS, fixed with cold ethanol for 25 min at −20 °C, and permeabilized with Triton X-100 (0.5%) in PBS for 15 min. Then, cells were blocked with normal serum (5%) in PBS for 1 h and incubated at 4 °C overnight with the two primary antibodies. A protocol previously described was applied [36], with the following antibodies: rabbit polyclonal anti human FZD 7 (diluted 1:200 in Blocking, Abcam, Cambridge, UK) and rabbit polyclonal anti human CB1R (diluted 1:200 in Blocking, Abcam, Cambridge, UK). After a wash, the cells were incubated with specific green fluorescent (goat antirabbit IgG (H + L) secondary antibody Alexa Fluor 488 conjugate) secondary antibodies for 1 h, at room temperature, in the dark. Subsequently, the cells were mounted using prolonged gold antifade reagent containing DAPI (blue). Images were acquired using the Nikon confocal microscope Eclipse Ti2, and the fluorescence intensity was quantified using Image J software (number of pixels/area). Five different areas from the single well for each single independent experiment performed in triplicate were randomly selected.

### 4.7. RNA Extraction and cDNA Synthesis

The RNA extraction was conducted from frozen cell pellets using PureLink^®^ RNA Mini Kit (Life technologies, 5791 Van Allen Way, Carlsband, CA, USA). All samples were stored at −80 °C before reverse transcription. The RNA concentration was measured using a NanoDrop Lite (Thermo Fisher Scientific). An aliquot of 2 μg of total RNA was transcribed by using iScript™ cDNA Synthesis kit (Bio-Rad, Hercules, CA, USA), following the reaction protocol: priming 5 min at 25 °C; reverse trasnscription 20 min at 46 °C; reverse transcription inactivation 1 min at 95 °C; and holding at 4 °C. The storage was −20 °C.

### 4.8. ddPCR Analysis

Copy numbers for microliter of *FZD 7*, *CNR1*, and *CNR2* cDNA were analyzed in treated HepaRG cells and quantified by droplet digital PCR (ddPCR; QX200 Droplet Digital PCR System, Bio-Rad, Hercules, CA, USA), according to the manufacturer’s instructions for EvaGreen protocol. The reaction was conducted with a total volume of 20 μL, including 15 ng of cDNA for sample, 10 μL of QX200™ ddPCR™ EvaGreen Supermix (Bio-Rad, Hercules, CA, USA), RNase-/DNase-free water (variable), and 100 nM primer SYBR^®^ Green Assay for ddPCR. The reagents were purchased from Bio-Rad with the following assay ID numbers: CNR1, qHsaCED0002285; CNR2, qHsaCED0038847. Cycling conditions were the following: 1 cycle at 95 °C for 5 min; 40 cycles at 95 °C for 30 s; 40 cycles at 60 °C for 1 min; 1 cycle at 4 °C for 5 min; 1 cycle at 90 °C for 5 min; holding at 4 °C. Data were processed using QX Manager 1.2 Standard Edition (BioRad).

### 4.9. IL-1β Assay

The levels of IL-1β in the colture medium of untreated and treated HepaRG cells for 72 h were measured with IL-1β ELISA kit (AbCam, Cambridge, UK) according to the manufacturer’s instructions.

### 4.10. Lipidomic Analysis

Total lipids were extracted from exosomal pellets and from untreated and treated HepaRG cell membranes. The Folch extraction method was used with some modifications [49]. Briefly, a 100 μL aliquot of cell lysate was diluted in 450 μL of an acidified saline solution (H2SO4 2 × 10^−4^ M, NaCl 0.1%). Subsequently, methanol:chloroform 1:2 was added. The lower layer containing the fatty acids was collected and evaporated using a centrifugal evaporator (Thermo Fisher Scientific, Waltham, MA, USA). The extracted fatty acids were converted to their corresponding fatty acid methyl esters (FAMEs) and analyzed in a gas chromatograph (Thermo Fisher Scientific, Focus GC, Milan, Italy) using ChromQuest 4.1 software (Thermo Fisher Scientific, Focus GC, Milan, Italy), as previously described [46,50]

### 4.11. Bioinformatic and Statistical Analysis

All gene and protein expression experiments of FZD 7, CB1R, CB2R, PPAR-γ, and IL-1β were reproduced 3 times. Bioinformatic interactions of the same genes were observed with the Search Tool for the Retrieval of Interacting Genes/Proteins (STRING) database, version 11.0. Statistical analysis was performed with Sigma-Stat 3.1 software, applying one-way analysis of variance (ANOVA) and Tukey’s post-test. The differences were considered statistically significant at *p* < 0.05.

## 5. Conclusions

The main findings of our study highlighted the presence of CB1R in the exosomes extracted from the plasma of NAFLD patients and its crucial role in the modulation of hepatic cells’ function. Despite the use of only one liver cell line, which could be a limitation of the study itself, our results shed light on a better understanding of the nature and secretion of circulating exosomes. Thus, the analysis of their loads may be useful in the diagnosis and treatment of liver diseases, including NAFLD.

## Figures and Tables

**Table 1:** IL-1β levels evaluated in the medium of control HepaRG cells (CTR), HepaRG cells treated with plasma-derived exosome from healthy subjects (H-EX), and NAFLD patients (P-EX) for 72 h.

	CTR	H-EX	P-EX
IL-1β (pg/mL)	19.11 ± 2.05	29.35 ± 3.452	66.83 ± 2.0568 **

Data expressed as mean value ± Standard Deviation (SD) of three consecutive experiments. *p*-value was determined by ANOVA with Tukey’s Multiple Comparison Test. ** *p* < 0.001.

**Table 2:** (A) Mean percentage of main saturated and monounsaturated fatty acids in exosomes derived by healthy subjects and NAFLD patients. (B) Mean percentage of main saturated and monounsaturated fatty acids in control untreated HepaRG cell membranes (CTR), HepaRG cell membranes treated with plasma-derived exosomes from healthy subjects (H-EX), and NAFLD patients (P-EX) for 24 h.

A	Exosome Fatty Acids (%)	Healthy Subjects	NAFLD Patients	
	Stearic acid (C18:0)	5.49 ± 0.41	6.26 ± 0.20 **	
	Oleic acid (C18:1n9)	26.18 ± 1.02	33.12 ± 3.87 **	
	Saturated fatty acids (SFAs)	30.72 ± 2.54	34.53 ± 2.60	
	Monounsaturated fatty acids (MUFAs)	32.53 ± 0.78	39.91 ± 3.17 **	
	Oleic acid/Stearic acid ratio (SCD-1)	4.78 ± 0.28	5.57 ± 0.63 *	
**B**	**HepaRG membrane Fatty Acids (%)**	**CTR**	**H-EX**	**P-EX**
	Stearic acid (C18:0)	10.07 ± 1.51	10.14 ± 1.70	11.07 ± 1.61
	Oleic acid (C18:1n9)	27.63 ± 1.13	27.92 ± 1.53	33.28 ± 1.31 ***
	Saturated fatty acids (SFAs)	32.48 ± 2.32	34.82 ± 1.09	35.93 ± 3.11
	Monounsaturated fatty acids (MUFAs)	49.63 ± 2.27	50.04 ± 2.14	56.21 ± 1.85 ***
	Oleic acid/Stearic acid ratio (SCD-1)	2.79 ± 0.41	2.80 ± 0.34	3.34 ± 0.25 *

Data expressed as mean value ± Standard Deviation (SD) of three consecutive experiments. *p*-value was determined by ANOVA with Tukey’s Multiple Comparison Test. * *p* < 0.05, ** *p* < 0.001, and *** *p* < 0.0001.

## Data Availability

The data used to support the findings of this study are included within the article.
